# Supplementary material for: An automated data processing and analysis pipeline for transmembrane proteins in detergent solutions
Source: Sci Rep. 2020 May 15;10:8081. doi: 10.1038/s41598-020-64933-1 (PMC7228933; doi:10.1038/s41598-020-64933-1)
Supplement: Supplementary file 1 — Supplementary Information. [file 41598_2020_64933_MOESM1_ESM.docx]

An automated data processing and analysis pipeline for transmembrane proteins in detergent solutions

D.Molodenskiy^1^, H. Mertens^1^ and D. Svergun^1*^

^1^ European Molecular Biology Laboratory (EMBL), Hamburg Unit, Notkestrasse

85, 22607, Hamburg, Germany

Corresponding author: [svergun@embl-hamburg.de](mailto:svergun@embl-hamburg.de)

**Supplementary information**


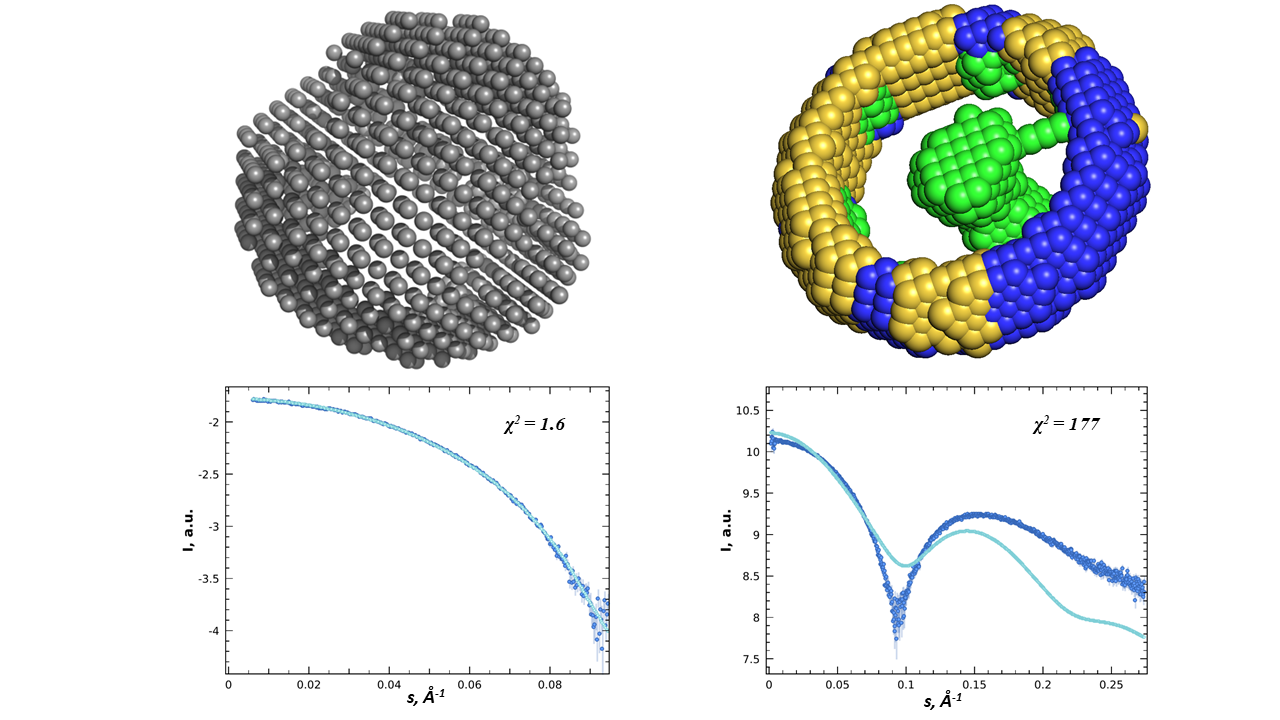


Figure. S1. For unloaded/empty detergent micelles, single phase DAMMIF reconstruction yields a hollow, close to spherical shape, fitting well the data at low angles (left panel). The multi-phase MONSA run requiring the presence of a phase corresponding to a protein component and the detergent belt confined to specific volumes will fail to adequately fit the data (right panel) providing the evidence that the data set is incompatible with the assumption made about the system.
